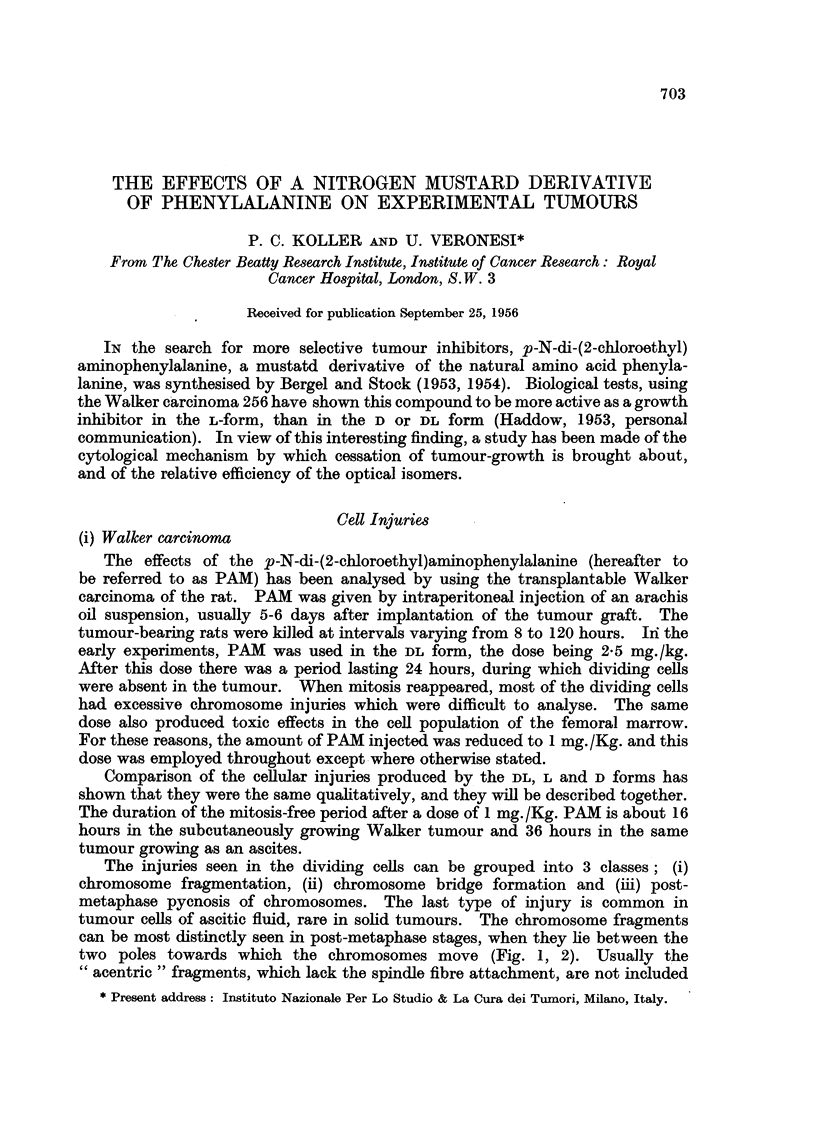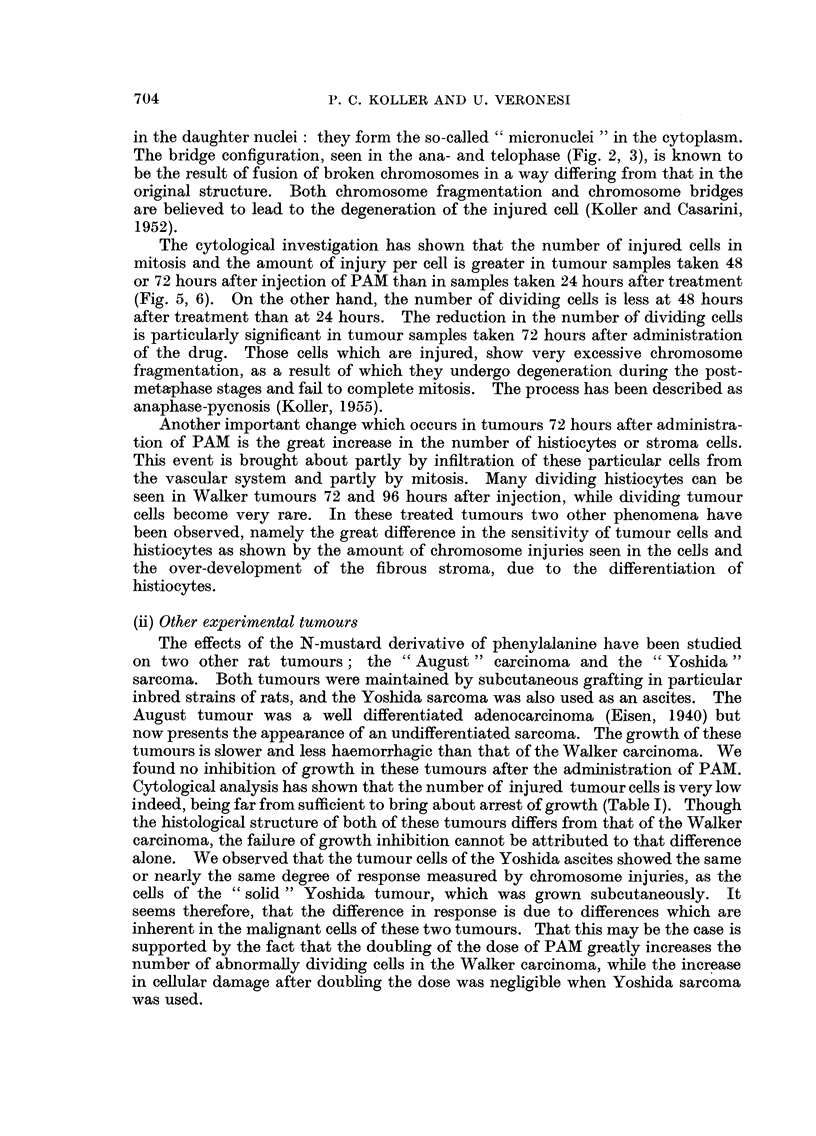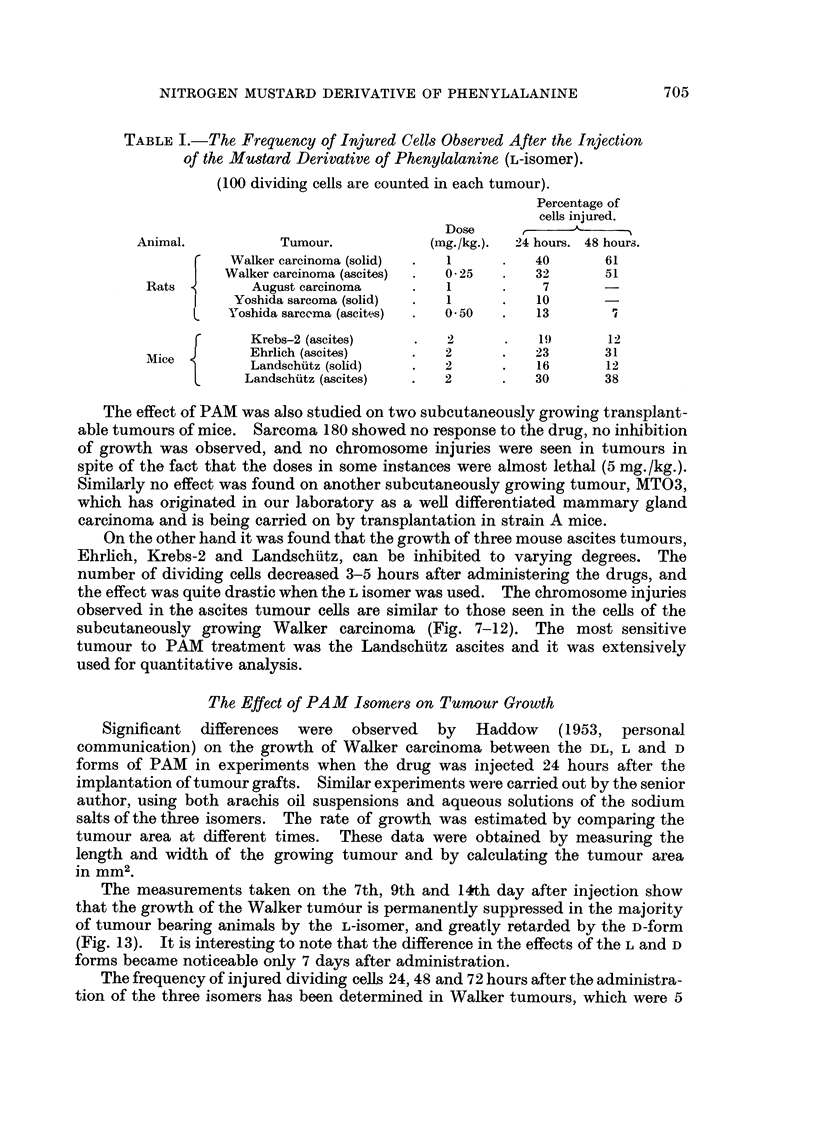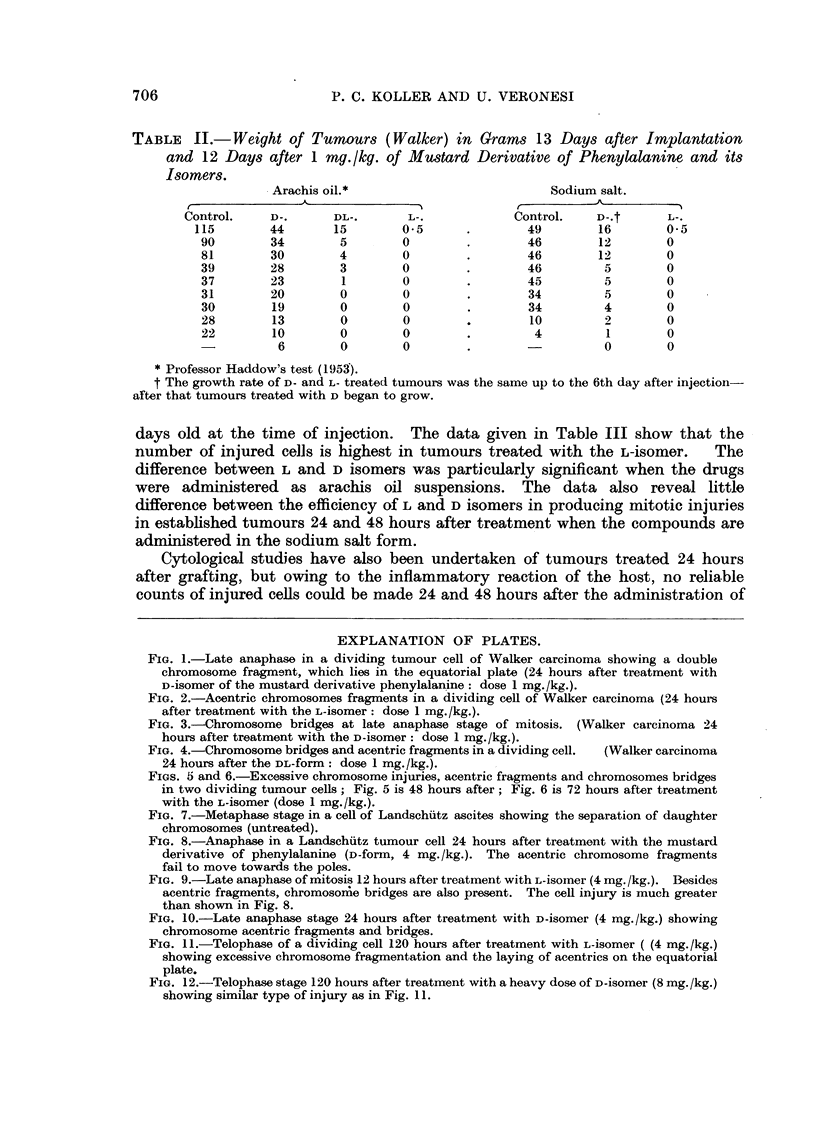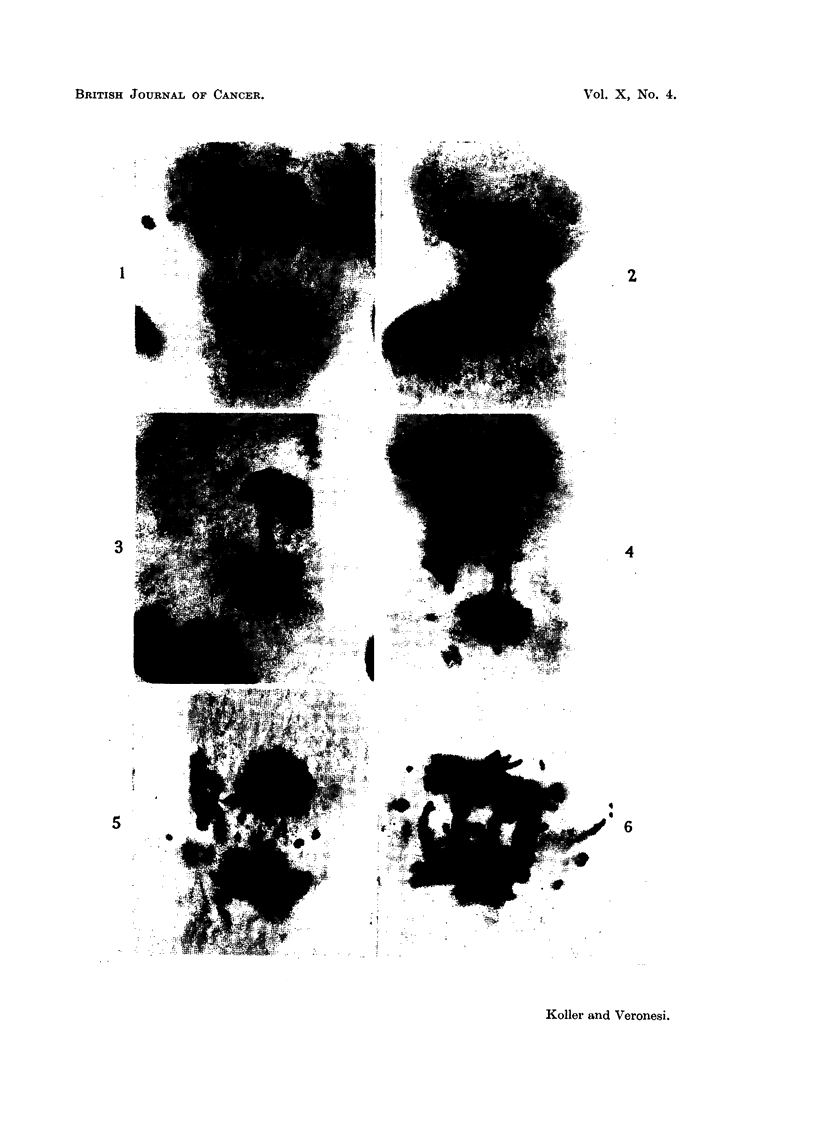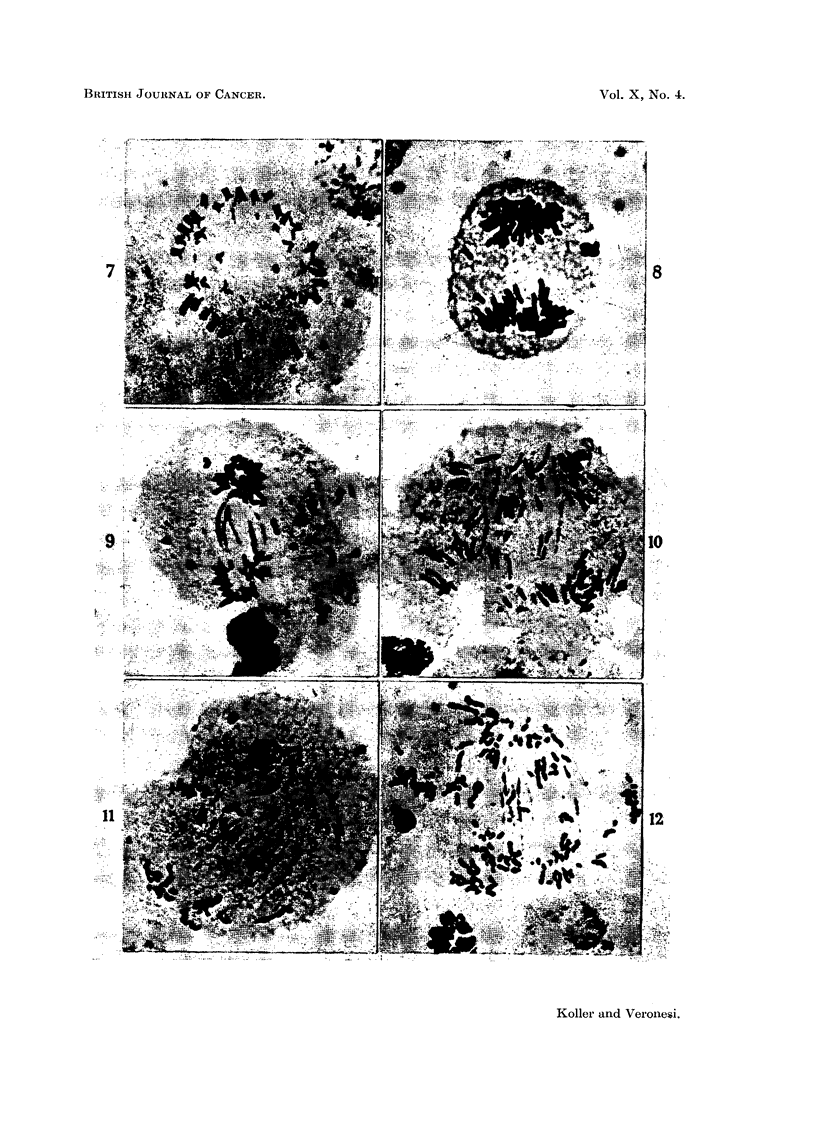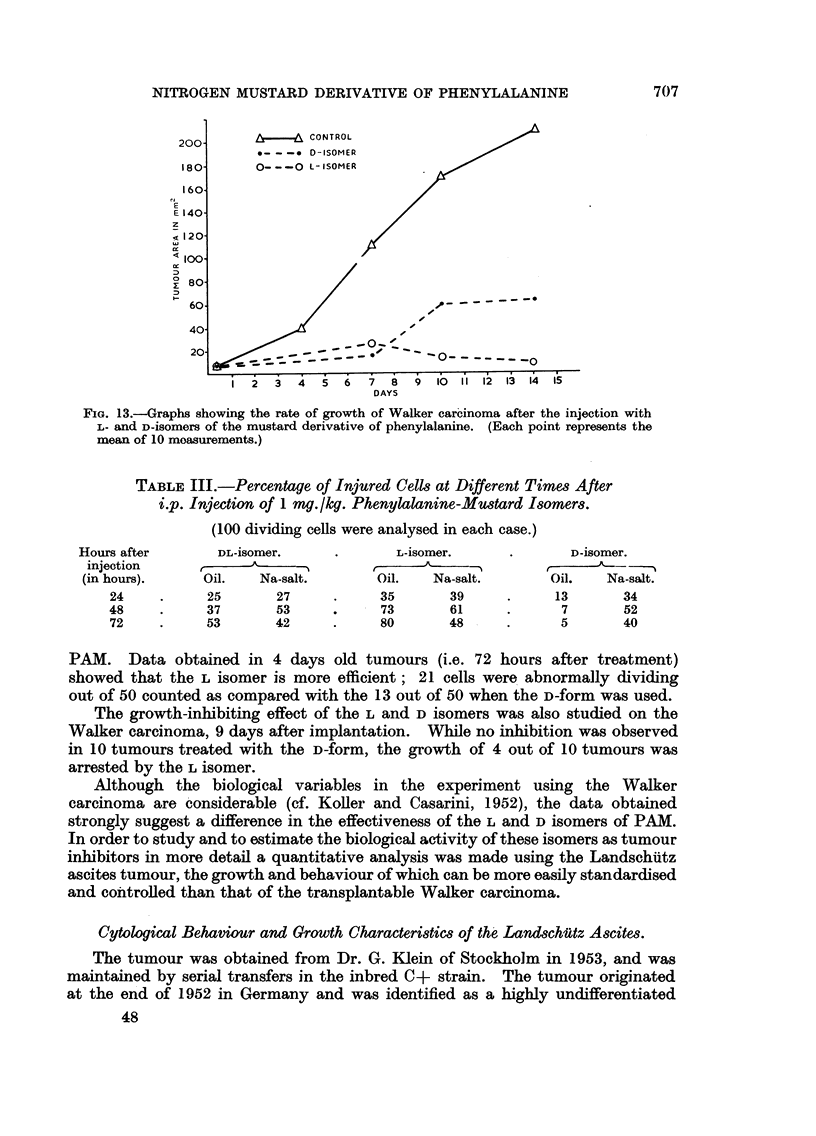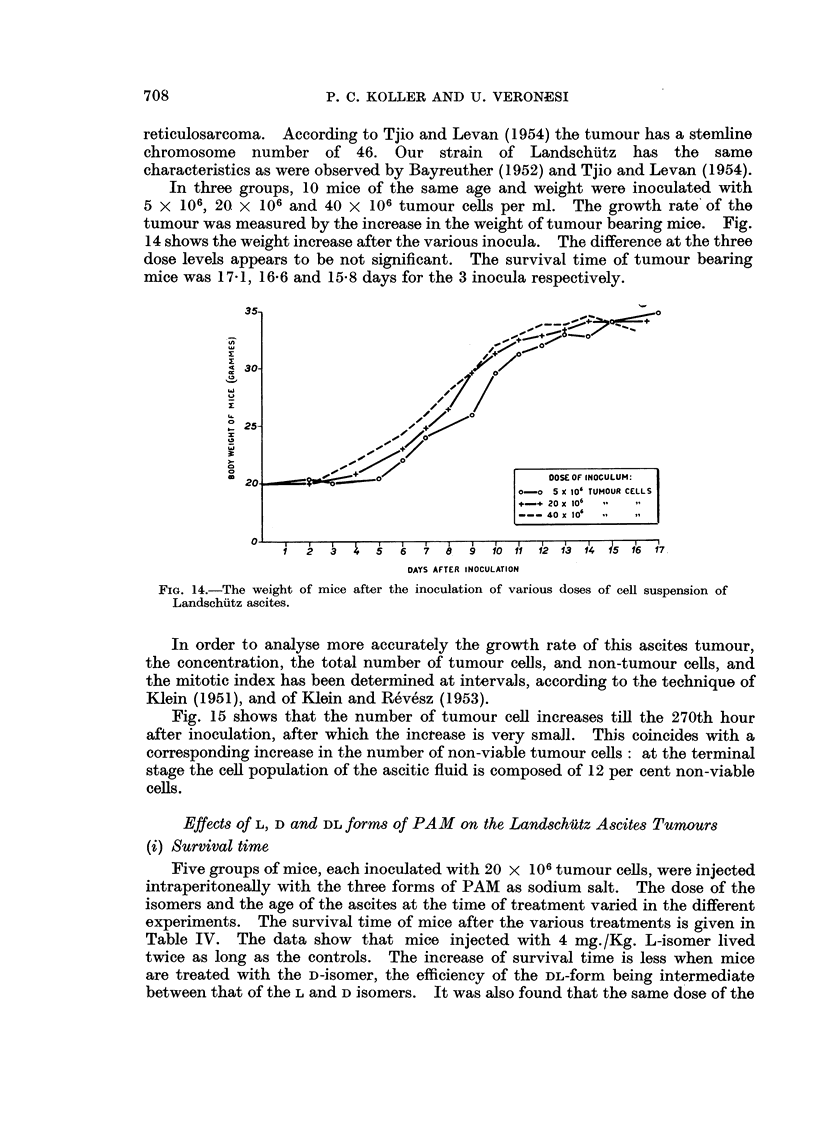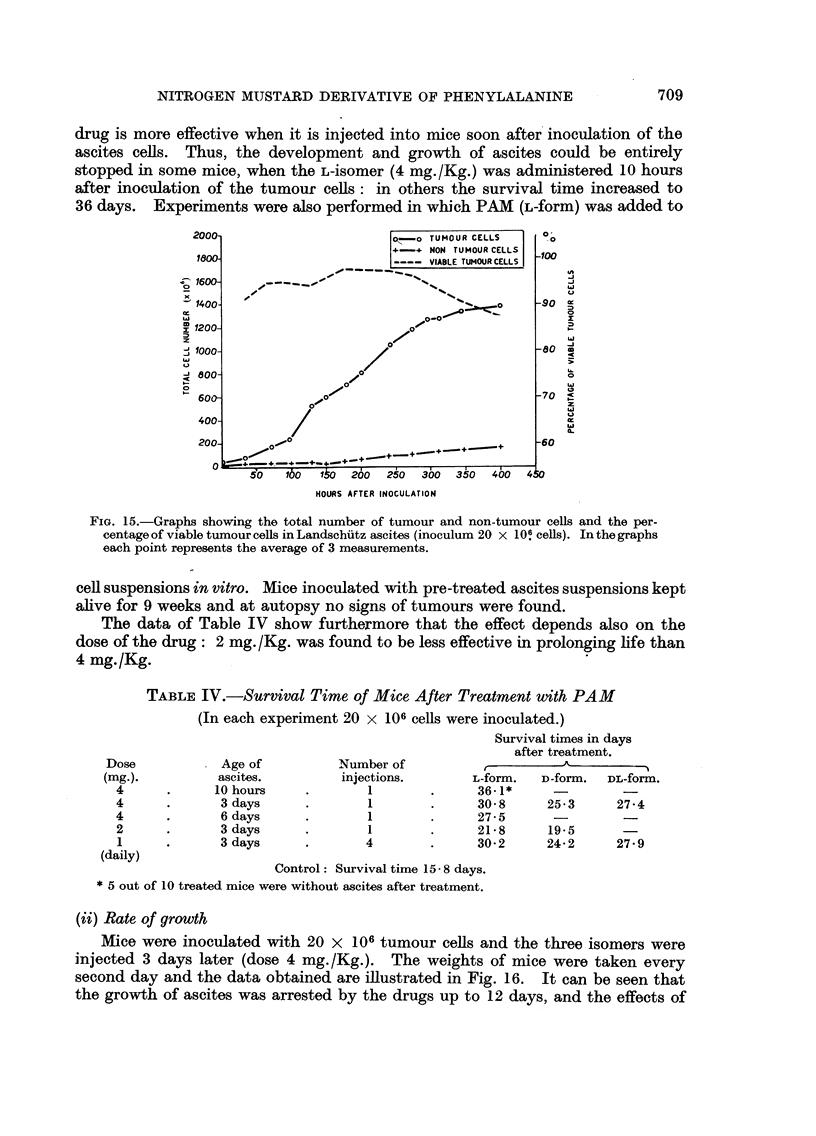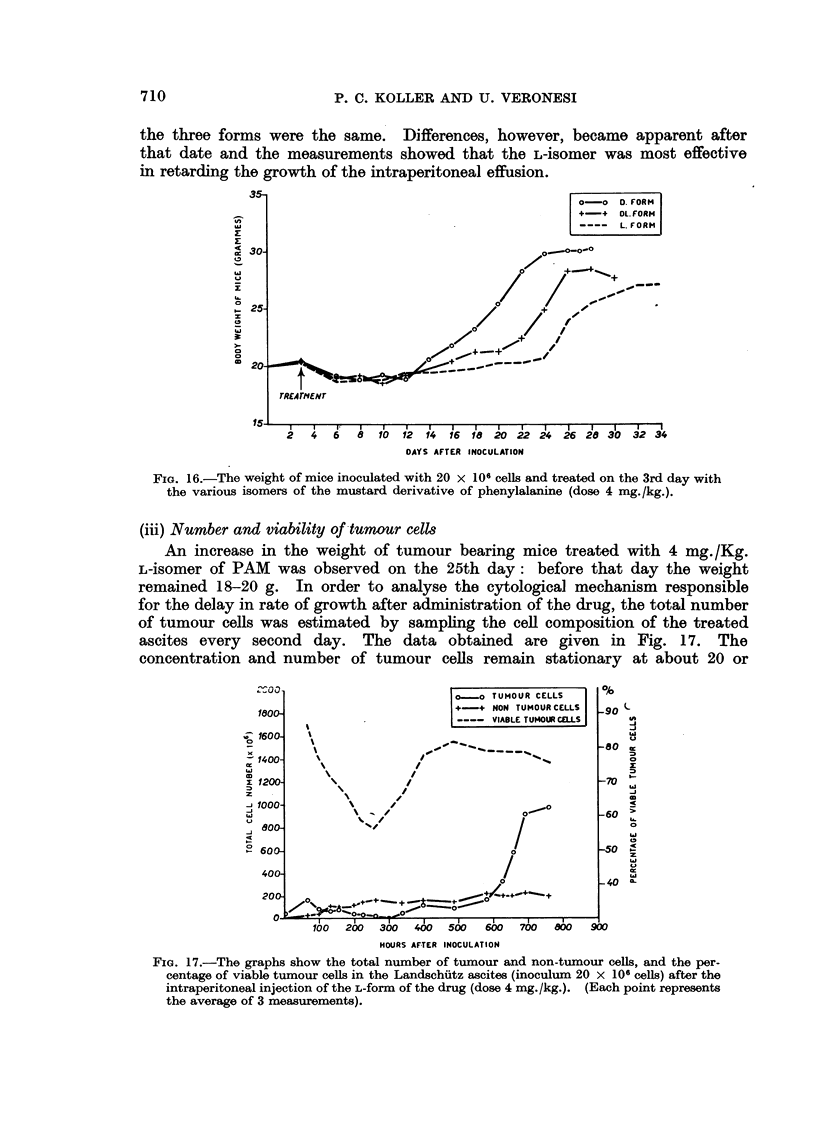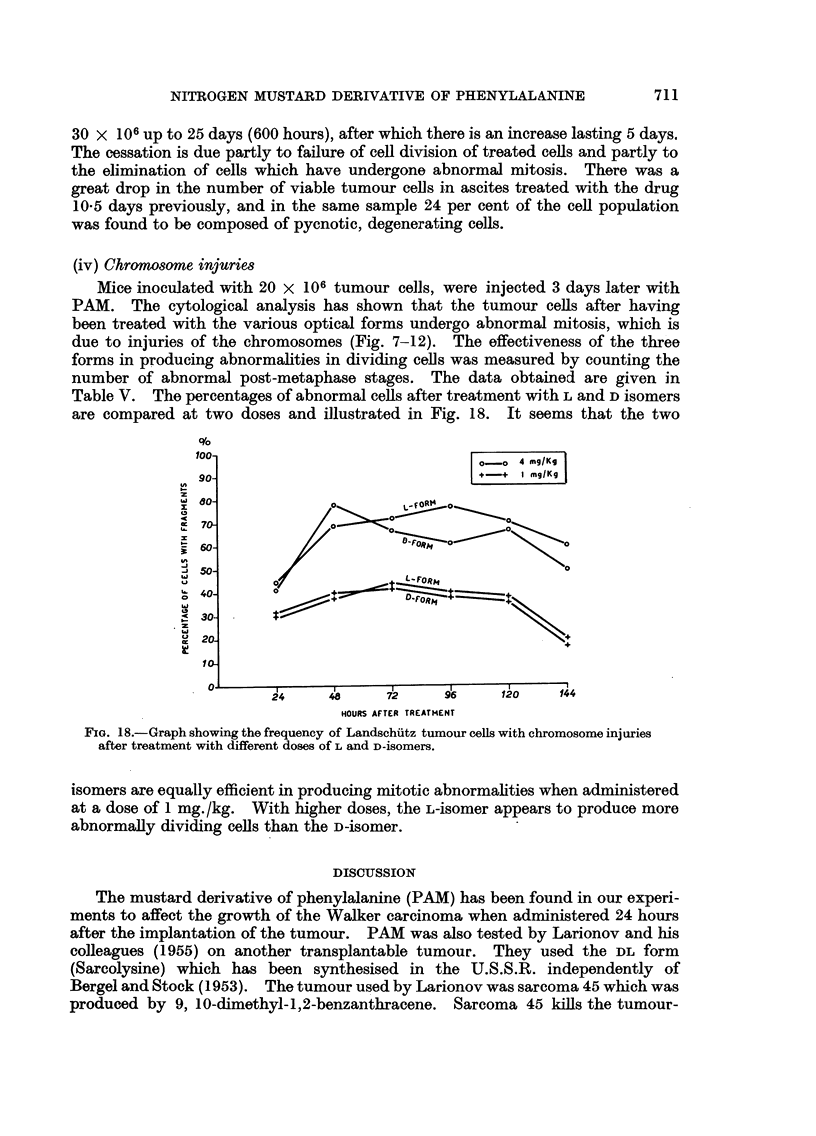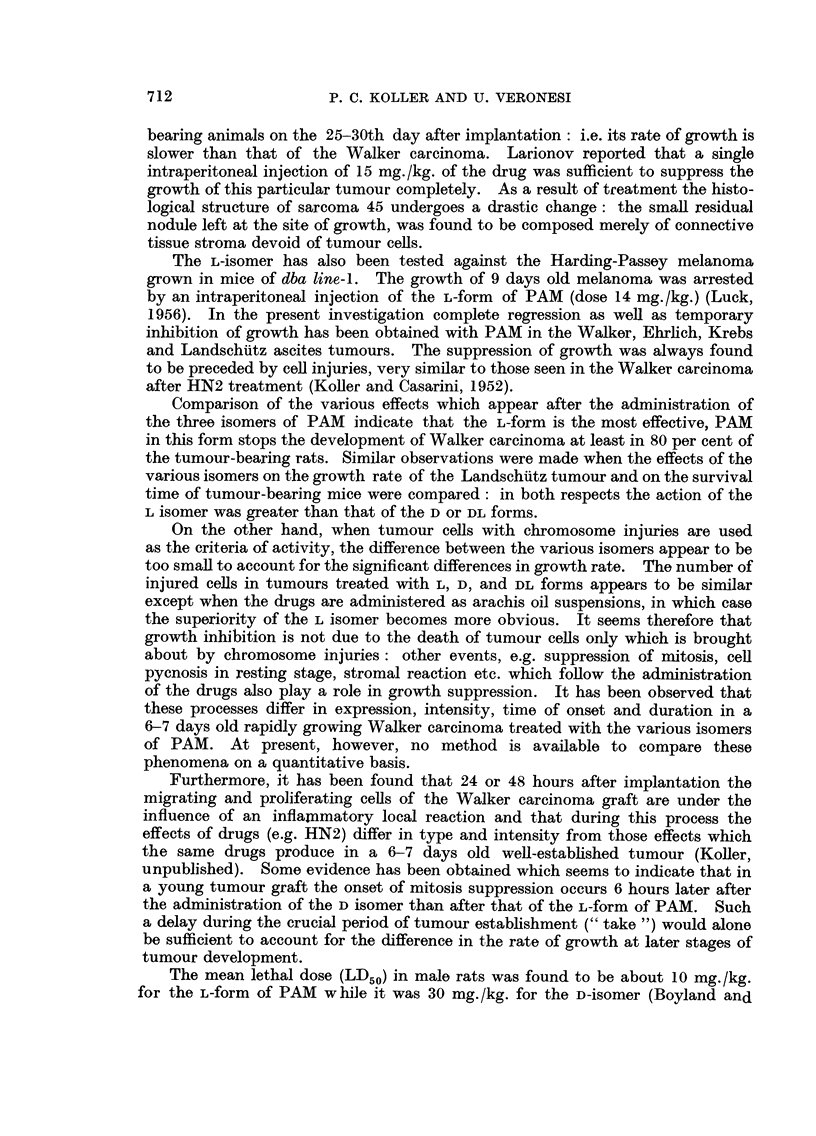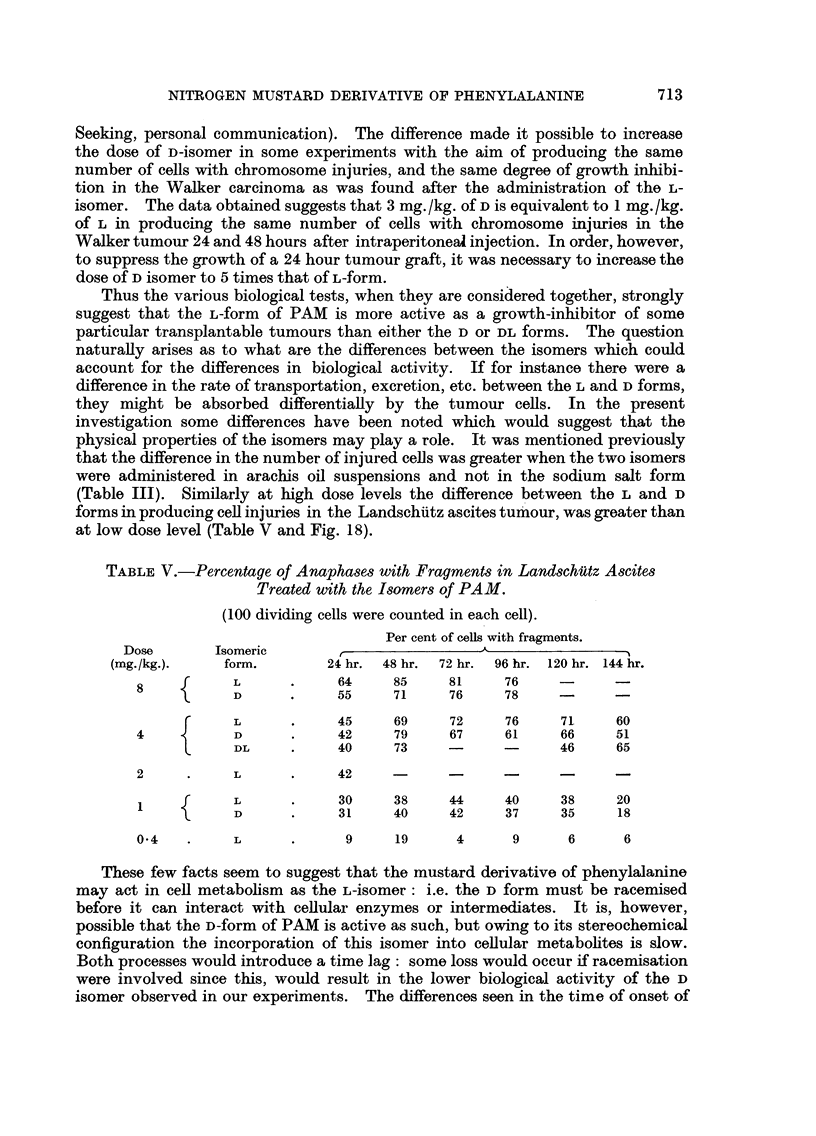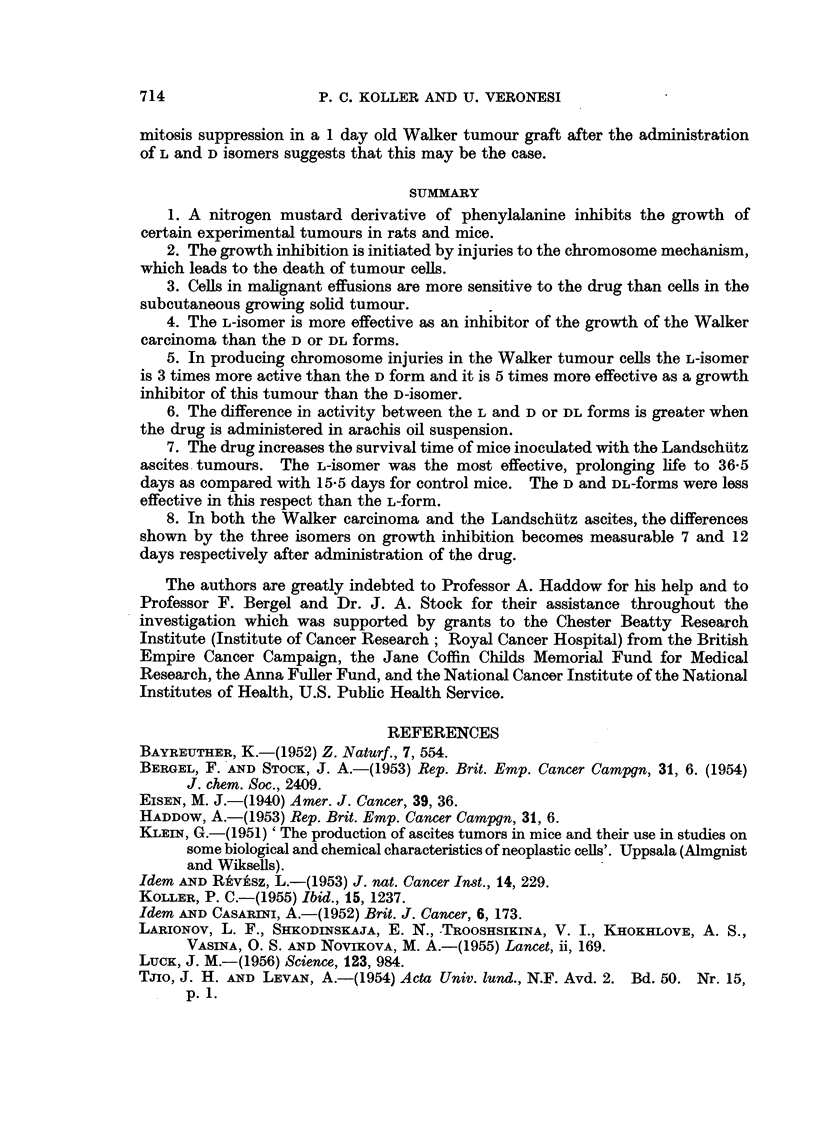# The Effects of a Nitrogen mustard Derivative of Phenylalanine on Experimental Tumours

**DOI:** 10.1038/bjc.1956.85

**Published:** 1956-12

**Authors:** P. C. Koller, U. Veronesi

## Abstract

**Images:**


### THE EFFECTS OF A NITROGEN MUSTARD DERIVATIVE OF PHENYLALANINE ON EXPERIMENTAL TUMOURS

P. C. KOLLER AND U. VERONESI*

*From The Chester Beatty Research Institute, Institute of Cancer Research: Royal Cancer Hospital, London, S.W. 3*

Received for publication September 25, 1956

IN the search for more selective tumour inhibitors, *p*-N-di-(2-chloroethyl) aminophenylalanine, a mustatd derivative of the natural amino acid phenylalanine, was synthesised by Bergel and Stock (1953, 1954). Biological tests, using the Walker carcinoma 256 have shown this compound to be more active as a growth inhibitor in the L-form, than in the D or DL form (Haddow, 1953, personal communication). In view of this interesting finding, a study has been made of the cytological mechanism by which cessation of tumour-growth is brought about, and of the relative efficiency of the optical isomers.

### *Cell Injuries*

### (i) *Walker carcinoma*

The effects of the *p*-N-di-(2-chloroethyl)aminophenylalanine (hereafter to be referred to as PAM) has been analysed by using the transplantable Walker carcinoma of the rat. PAM was given by intraperitoneal injection of an arachis oil suspension, usually 5-6 days after implantation of the tumour graft. The tumour-bearing rats were killed at intervals varying from 8 to 120 hours. In the early experiments, PAM was used in the DL form, the dose being 2·5 mg./kg. After this dose there was a period lasting 24 hours, during which dividing cells were absent in the tumour. When mitosis reappeared, most of the dividing cells had excessive chromosome injuries which were difficult to analyse. The same dose also produced toxic effects in the cell population of the femoral marrow. For these reasons, the amount of PAM injected was reduced to 1 mg./Kg. and this dose was employed throughout except where otherwise stated.

Comparison of the cellular injuries produced by the DL, L and D forms has shown that they were the same qualitatively, and they will be described together. The duration of the mitosis-free period after a dose of 1 mg./Kg. PAM is about 16 hours in the subcutaneously growing Walker tumour and 36 hours in the same tumour growing as an ascites.

The injuries seen in the dividing cells can be grouped into 3 classes; (i) chromosome fragmentation, (ii) chromosome bridge formation and (iii) post-metaphase pycnosis of chromosomes. The last type of injury is common in tumour cells of ascitic fluid, rare in solid tumours. The chromosome fragments can be most distinctly seen in post-metaphase stages, when they lie between the two poles towards which the chromosomes move (Fig. 1, 2). Usually the "acentric" fragments, which lack the spindle fibre attachment, are not included

* Present address: Instituto Nazionale Per Lo Studio & La Cura dei Tumori, Milano, Italy.

in the daughter nuclei : they form the so-called " micronuclei " in the cytoplasm. The bridge configuration, seen in the ana- and telophase (Fig. 2, 3), is known to be the result of fusion of broken chromosomes in a way differing from that in the original structure. Both chromosome fragmentation and chromosome bridges are believed to lead to the degeneration of the injured cell (Koller and Casarini, 1952).

The cytological investigation has shown that the number of injured cells in mitosis and the amount of injury per cell is greater in tumour samples taken 48 or 72 hours after injection of PAM than in samples taken 24 hours after treatment (Fig. 5, 6). On the other hand, the number of dividing cells is less at 48 hours after treatment than at 24 hours. The reduction in the number of dividing cells is particularly significant in tumour samples taken 72 hours after administration of the drug. Those cells which are injured, show very excessive chromosome fragmentation, as a result of which they undergo degeneration during the post-metaphase stages and fail to complete mitosis. The process has been described as anaphase-pycnosis (Koller, 1955).

Another important change which occurs in tumours 72 hours after administration of PAM is the great increase in the number of histiocytes or stroma cells. This event is brought about partly by infiltration of these particular cells from the vascular system and partly by mitosis. Many dividing histiocytes can be seen in Walker tumours 72 and 96 hours after injection, while dividing tumour cells become very rare. In these treated tumours two other phenomena have been observed, namely the great difference in the sensitivity of tumour cells and histiocytes as shown by the amount of chromosome injuries seen in the cells and the over-development of the fibrous stroma, due to the differentiation of histiocytes.

(ii) *Other experimental tumours*

The effects of the N-mustard derivative of phenylalanine have been studied on two other rat tumours ; the " August " carcinoma and the " Yoshida " sarcoma. Both tumours were maintained by subcutaneous grafting in particular inbred strains of rats, and the Yoshida sarcoma was also used as an ascites. The August tumour was a well differentiated adenocarcinoma (Eisen, 1940) but now presents the appearance of an undifferentiated sarcoma. The growth of these tumours is slower and less haemorrhagic than that of the Walker carcinoma. We found no inhibition of growth in these tumours after the administration of PAM. Cytological analysis has shown that the number of injured tumour cells is very low indeed, being far from sufficient to bring about arrest of growth (Table I). Though the histological structure of both of these tumours differs from that of the Walker carcinoma, the failure of growth inhibition cannot be attributed to that difference alone. We observed that the tumour cells of the Yoshida ascites showed the same or nearly the same degree of response measured by chromosome injuries, as the cells of the " solid " Yoshida tumour, which was grown subcutaneously. It seems therefore, that the difference in response is due to differences which are inherent in the malignant cells of these two tumours. That this may be the case is supported by the fact that the doubling of the dose of PAM greatly increases the number of abnormally dividing cells in the Walker carcinoma, while the increase in cellular damage after doubling the dose was negligible when Yoshida sarcoma was used.

TABLE I.—*The Frequency of Injured Cells Observed After the Injection of the Mustard Derivative of Phenylalanine* (L-isomer).

(100 dividing cells are counted in each tumour).

| Animal. | Tumour. | Dose (mg./kg.). | Percentage of cells injured. 24 hours. | 48 hours. |
|---|---|---|---|---|
| Rats | Walker carcinoma (solid) | 1 | 40 | 61 |
| | Walker carcinoma (ascites) | 0·25 | 32 | 51 |
| | August carcinoma | 1 | 7 | — |
| | Yoshida sarcoma (solid) | 1 | 10 | — |
| | Yoshida sarcoma (ascites) | 0·50 | 13 | 7 |
| Mice | Krebs-2 (ascites) | 2 | 19 | 12 |
| | Ehrlich (ascites) | 2 | 23 | 31 |
| | Landschütz (solid) | 2 | 16 | 12 |
| | Landschütz (ascites) | 2 | 30 | 38 |

The effect of PAM was also studied on two subcutaneously growing transplantable tumours of mice. Sarcoma 180 showed no response to the drug, no inhibition of growth was observed, and no chromosome injuries were seen in tumours in spite of the fact that the doses in some instances were almost lethal (5 mg./kg.). Similarly no effect was found on another subcutaneously growing tumour, MTO3, which has originated in our laboratory as a well differentiated mammary gland carcinoma and is being carried on by transplantation in strain A mice.

On the other hand it was found that the growth of three mouse ascites tumours, Ehrlich, Krebs-2 and Landschütz, can be inhibited to varying degrees. The number of dividing cells decreased 3–5 hours after administering the drugs, and the effect was quite drastic when the L isomer was used. The chromosome injuries observed in the ascites tumour cells are similar to those seen in the cells of the subcutaneously growing Walker carcinoma (Fig. 7–12). The most sensitive tumour to PAM treatment was the Landschütz ascites and it was extensively used for quantitative analysis.

### *The Effect of PAM Isomers on Tumour Growth*

Significant differences were observed by Haddow (1953, personal communication) on the growth of Walker carcinoma between the DL, L and D forms of PAM in experiments when the drug was injected 24 hours after the implantation of tumour grafts. Similar experiments were carried out by the senior author, using both arachis oil suspensions and aqueous solutions of the sodium salts of the three isomers. The rate of growth was estimated by comparing the tumour area at different times. These data were obtained by measuring the length and width of the growing tumour and by calculating the tumour area in $mm^2$.

The measurements taken on the 7th, 9th and 14th day after injection show that the growth of the Walker tumour is permanently suppressed in the majority of tumour bearing animals by the L-isomer, and greatly retarded by the D-form (Fig. 13). It is interesting to note that the difference in the effects of the L and D forms became noticeable only 7 days after administration.

The frequency of injured dividing cells 24, 48 and 72 hours after the administration of the three isomers has been determined in Walker tumours, which were 5

TABLE II.—*Weight of Tumours (Walker) in Grams 13 Days after Implantation and 12 Days after 1 mg./kg. of Mustard Derivative of Phenylalanine and its Isomers.*

| Arachis oil.* | | | | Sodium salt. | | |
|---|---|---|---|---|---|---|
| Control. | D-. | DL-. | L-. | Control. | D-.† | L-. |
| 115 | 44 | 15 | 0·5 | 49 | 16 | 0·5 |
| 90 | 34 | 5 | 0 | 46 | 12 | 0 |
| 81 | 30 | 4 | 0 | 46 | 12 | 0 |
| 39 | 28 | 3 | 0 | 46 | 5 | 0 |
| 37 | 23 | 1 | 0 | 45 | 5 | 0 |
| 31 | 20 | 0 | 0 | 34 | 5 | 0 |
| 30 | 19 | 0 | 0 | 34 | 4 | 0 |
| 28 | 13 | 0 | 0 | 10 | 2 | 0 |
| 22 | 10 | 0 | 0 | 4 | 1 | 0 |
| — | 6 | 0 | 0 | — | 0 | 0 |

* Professor Haddow's test (1953).
† The growth rate of D- and L- treated tumours was the same up to the 6th day after injection—after that tumours treated with D began to grow.

days old at the time of injection. The data given in Table III show that the number of injured cells is highest in tumours treated with the L-isomer. The difference between L and D isomers was particularly significant when the drugs were administered as arachis oil suspensions. The data also reveal little difference between the efficiency of L and D isomers in producing mitotic injuries in established tumours 24 and 48 hours after treatment when the compounds are administered in the sodium salt form.

Cytological studies have also been undertaken of tumours treated 24 hours after grafting, but owing to the inflammatory reaction of the host, no reliable counts of injured cells could be made 24 and 48 hours after the administration of

### EXPLANATION OF PLATES.

FIG. 1.—Late anaphase in a dividing tumour cell of Walker carcinoma showing a double chromosome fragment, which lies in the equatorial plate (24 hours after treatment with D-isomer of the mustard derivative phenylalanine : dose 1 mg./kg.).

FIG. 2.—Acentric chromosomes fragments in a dividing cell of Walker carcinoma (24 hours after treatment with the L-isomer : dose 1 mg./kg.).

FIG. 3.—Chromosome bridges at late anaphase stage of mitosis. (Walker carcinoma 24 hours after treatment with the D-isomer : dose 1 mg./kg.).

FIG. 4.—Chromosome bridges and acentric fragments in a dividing cell. (Walker carcinoma 24 hours after the DL-form : dose 1 mg./kg.).

FIGS. 5 and 6.—Excessive chromosome injuries, acentric fragments and chromosomes bridges in two dividing tumour cells ; Fig. 5 is 48 hours after ; Fig. 6 is 72 hours after treatment with the L-isomer (dose 1 mg./kg.).

FIG. 7.—Metaphase stage in a cell of Landschütz ascites showing the separation of daughter chromosomes (untreated).

FIG. 8.—Anaphase in a Landschütz tumour cell 24 hours after treatment with the mustard derivative of phenylalanine (D-form, 4 mg./kg.). The acentric chromosome fragments fail to move towards the poles.

FIG. 9.—Late anaphase of mitosis 12 hours after treatment with L-isomer (4 mg./kg.). Besides acentric fragments, chromosome bridges are also present. The cell injury is much greater than shown in Fig. 8.

FIG. 10.—Late anaphase stage 24 hours after treatment with D-isomer (4 mg./kg.) showing chromosome acentric fragments and bridges.

FIG. 11.—Telophase of a dividing cell 120 hours after treatment with L-isomer ( (4 mg./kg.) showing excessive chromosome fragmentation and the laying of acentrics on the equatorial plate.

FIG. 12.—Telophase stage 120 hours after treatment with a heavy dose of D-isomer (8 mg./kg.) showing similar type of injury as in Fig. 11.

1
2
3
4
5
6

7

8

9

10

11

12

FIG. 13.—Graphs showing the rate of growth of Walker carcinoma after the injection with L- and D-isomers of the mustard derivative of phenylalanine. (Each point represents the mean of 10 measurements.)

TABLE III.—*Percentage of Injured Cells at Different Times After i.p. Injection of* 1 *mg./kg. Phenylalanine-Mustard Isomers.*

(100 dividing cells were analysed in each case.)

| Hours after injection (in hours). | DL-isomer. Oil. | DL-isomer. Na-salt. | L-isomer. Oil. | L-isomer. Na-salt. | D-isomer. Oil. | D-isomer. Na-salt. |
|---|---|---|---|---|---|---|
| 24 | 25 | 27 | 35 | 39 | 13 | 34 |
| 48 | 37 | 53 | 73 | 61 | 7 | 52 |
| 72 | 53 | 42 | 80 | 48 | 5 | 40 |

PAM. Data obtained in 4 days old tumours (i.e. 72 hours after treatment) showed that the L isomer is more efficient ; 21 cells were abnormally dividing out of 50 counted as compared with the 13 out of 50 when the D-form was used.

The growth-inhibiting effect of the L and D isomers was also studied on the Walker carcinoma, 9 days after implantation. While no inhibition was observed in 10 tumours treated with the D-form, the growth of 4 out of 10 tumours was arrested by the L isomer.

Although the biological variables in the experiment using the Walker carcinoma are considerable (cf. Koller and Casarini, 1952), the data obtained strongly suggest a difference in the effectiveness of the L and D isomers of PAM. In order to study and to estimate the biological activity of these isomers as tumour inhibitors in more detail a quantitative analysis was made using the Landschütz ascites tumour, the growth and behaviour of which can be more easily standardised and controlled than that of the transplantable Walker carcinoma.

*Cytological Behaviour and Growth Characteristics of the Landschütz Ascites.*

The tumour was obtained from Dr. G. Klein of Stockholm in 1953, and was maintained by serial transfers in the inbred C+ strain. The tumour originated at the end of 1952 in Germany and was identified as a highly undifferentiated reticulosarcoma. According to Tjio and Levan (1954) the tumour has a stemline chromosome number of 46. Our strain of Landschütz has the same characteristics as were observed by Bayreuther (1952) and Tjio and Levan (1954).

In three groups, 10 mice of the same age and weight were inoculated with $5 \times 10^6$, $20 \times 10^6$ and $40 \times 10^6$ tumour cells per ml. The growth rate of the tumour was measured by the increase in the weight of tumour bearing mice. Fig. 14 shows the weight increase after the various inocula. The difference at the three dose levels appears to be not significant. The survival time of tumour bearing mice was 17·1, 16·6 and 15·8 days for the 3 inocula respectively.

FIG. 14.—The weight of mice after the inoculation of various doses of cell suspension of Landschütz ascites.

In order to analyse more accurately the growth rate of this ascites tumour, the concentration, the total number of tumour cells, and non-tumour cells, and the mitotic index has been determined at intervals, according to the technique of Klein (1951), and of Klein and Révész (1953).

Fig. 15 shows that the number of tumour cell increases till the 270th hour after inoculation, after which the increase is very small. This coincides with a corresponding increase in the number of non-viable tumour cells : at the terminal stage the cell population of the ascitic fluid is composed of 12 per cent non-viable cells.

### *Effects of* L, D *and* DL *forms of PAM on the Landschütz Ascites Tumours*

*(i) Survival time*

Five groups of mice, each inoculated with $20 \times 10^6$ tumour cells, were injected intraperitoneally with the three forms of PAM as sodium salt. The dose of the isomers and the age of the ascites at the time of treatment varied in the different experiments. The survival time of mice after the various treatments is given in Table IV. The data show that mice injected with 4 mg./Kg. L-isomer lived twice as long as the controls. The increase of survival time is less when mice are treated with the D-isomer, the efficiency of the DL-form being intermediate between that of the L and D isomers. It was also found that the same dose of the drug is more effective when it is injected into mice soon after inoculation of the ascites cells. Thus, the development and growth of ascites could be entirely stopped in some mice, when the L-isomer (4 mg./Kg.) was administered 10 hours after inoculation of the tumour cells : in others the survival time increased to 36 days. Experiments were also performed in which PAM (L-form) was added to

FIG. 15.—Graphs showing the total number of tumour and non-tumour cells and the percentage of viable tumour cells in Landschütz ascites (inoculum 20 × $10^6$ cells). In the graphs each point represents the average of 3 measurements.

cell suspensions *in vitro*. Mice inoculated with pre-treated ascites suspensions kept alive for 9 weeks and at autopsy no signs of tumours were found.

The data of Table IV show furthermore that the effect depends also on the dose of the drug : 2 mg./Kg. was found to be less effective in prolonging life than 4 mg./Kg.

TABLE IV.—*Survival Time of Mice After Treatment with PAM*

(In each experiment 20 × $10^6$ cells were inoculated.)

| Dose (mg.). | Age of ascites. | Number of injections. | Survival times in days after treatment. L-form. | D-form. | DL-form. |
|---|---|---|---|---|---|
| 4 | 10 hours | 1 | 36·1* | — | — |
| 4 | 3 days | 1 | 30·8 | 25·3 | 27·4 |
| 4 | 6 days | 1 | 27·5 | — | — |
| 2 | 3 days | 1 | 21·8 | 19·5 | — |
| 1 (daily) | 3 days | 4 | 30·2 | 24·2 | 27·9 |

Control : Survival time 15·8 days.

* 5 out of 10 treated mice were without ascites after treatment.

*(ii) Rate of growth*

Mice were inoculated with 20 × $10^6$ tumour cells and the three isomers were injected 3 days later (dose 4 mg./Kg.). The weights of mice were taken every second day and the data obtained are illustrated in Fig. 16. It can be seen that the growth of ascites was arrested by the drugs up to 12 days, and the effects of the three forms were the same. Differences, however, became apparent after that date and the measurements showed that the L-isomer was most effective in retarding the growth of the intraperitoneal effusion.

FIG. 16.—The weight of mice inoculated with 20 × $10^6$ cells and treated on the 3rd day with the various isomers of the mustard derivative of phenylalanine (dose 4 mg./kg.).

(iii) *Number and viability of tumour cells*

An increase in the weight of tumour bearing mice treated with 4 mg./Kg. L-isomer of PAM was observed on the 25th day : before that day the weight remained 18–20 g. In order to analyse the cytological mechanism responsible for the delay in rate of growth after administration of the drug, the total number of tumour cells was estimated by sampling the cell composition of the treated ascites every second day. The data obtained are given in Fig. 17. The concentration and number of tumour cells remain stationary at about 20 or

FIG. 17.—The graphs show the total number of tumour and non-tumour cells, and the percentage of viable tumour cells in the Landschütz ascites (inoculum 20 × $10^6$ cells) after the intraperitoneal injection of the L-form of the drug (dose 4 mg./kg.). (Each point represents the average of 3 measurements).

$30 \times 10^6$ up to 25 days (600 hours), after which there is an increase lasting 5 days. The cessation is due partly to failure of cell division of treated cells and partly to the elimination of cells which have undergone abnormal mitosis. There was a great drop in the number of viable tumour cells in ascites treated with the drug 10·5 days previously, and in the same sample 24 per cent of the cell population was found to be composed of pycnotic, degenerating cells.

(iv) *Chromosome injuries*

Mice inoculated with $20 \times 10^6$ tumour cells, were injected 3 days later with PAM. The cytological analysis has shown that the tumour cells after having been treated with the various optical forms undergo abnormal mitosis, which is due to injuries of the chromosomes (Fig. 7–12). The effectiveness of the three forms in producing abnormalities in dividing cells was measured by counting the number of abnormal post-metaphase stages. The data obtained are given in Table V. The percentages of abnormal cells after treatment with L and D isomers are compared at two doses and illustrated in Fig. 18. It seems that the two

FIG. 18.—Graph showing the frequency of Landschütz tumour cells with chromosome injuries after treatment with different doses of L and D-isomers.

isomers are equally efficient in producing mitotic abnormalities when administered at a dose of 1 mg./kg. With higher doses, the L-isomer appears to produce more abnormally dividing cells than the D-isomer.

DISCUSSION

The mustard derivative of phenylalanine (PAM) has been found in our experiments to affect the growth of the Walker carcinoma when administered 24 hours after the implantation of the tumour. PAM was also tested by Larionov and his colleagues (1955) on another transplantable tumour. They used the DL form (Sarcolysine) which has been synthesised in the U.S.S.R. independently of Bergel and Stock (1953). The tumour used by Larionov was sarcoma 45 which was produced by 9, 10-dimethyl-1,2-benzanthracene. Sarcoma 45 kills the tumourbearing animals on the 25–30th day after implantation : i.e. its rate of growth is slower than that of the Walker carcinoma. Larionov reported that a single intraperitoneal injection of 15 mg./kg. of the drug was sufficient to suppress the growth of this particular tumour completely. As a result of treatment the histological structure of sarcoma 45 undergoes a drastic change : the small residual nodule left at the site of growth, was found to be composed merely of connective tissue stroma devoid of tumour cells.

The L-isomer has also been tested against the Harding-Passey melanoma grown in mice of *dba line*-1. The growth of 9 days old melanoma was arrested by an intraperitoneal injection of the L-form of PAM (dose 14 mg./kg.) (Luck, 1956). In the present investigation complete regression as well as temporary inhibition of growth has been obtained with PAM in the Walker, Ehrlich, Krebs and Landschütz ascites tumours. The suppression of growth was always found to be preceded by cell injuries, very similar to those seen in the Walker carcinoma after HN2 treatment (Koller and Casarini, 1952).

Comparison of the various effects which appear after the administration of the three isomers of PAM indicate that the L-form is the most effective, PAM in this form stops the development of Walker carcinoma at least in 80 per cent of the tumour-bearing rats. Similar observations were made when the effects of the various isomers on the growth rate of the Landschütz tumour and on the survival time of tumour-bearing mice were compared : in both respects the action of the L isomer was greater than that of the D or DL forms.

On the other hand, when tumour cells with chromosome injuries are used as the criteria of activity, the difference between the various isomers appear to be too small to account for the significant differences in growth rate. The number of injured cells in tumours treated with L, D, and DL forms appears to be similar except when the drugs are administered as arachis oil suspensions, in which case the superiority of the L isomer becomes more obvious. It seems therefore that growth inhibition is not due to the death of tumour cells only which is brought about by chromosome injuries : other events, e.g. suppression of mitosis, cell pycnosis in resting stage, stromal reaction etc. which follow the administration of the drugs also play a role in growth suppression. It has been observed that these processes differ in expression, intensity, time of onset and duration in a 6–7 days old rapidly growing Walker carcinoma treated with the various isomers of PAM. At present, however, no method is available to compare these phenomena on a quantitative basis.

Furthermore, it has been found that 24 or 48 hours after implantation the migrating and proliferating cells of the Walker carcinoma graft are under the influence of an inflammatory local reaction and that during this process the effects of drugs (e.g. HN2) differ in type and intensity from those effects which the same drugs produce in a 6–7 days old well-established tumour (Koller, unpublished). Some evidence has been obtained which seems to indicate that in a young tumour graft the onset of mitosis suppression occurs 6 hours later after the administration of the D isomer than after that of the L-form of PAM. Such a delay during the crucial period of tumour establishment (" take ") would alone be sufficient to account for the difference in the rate of growth at later stages of tumour development.

The mean lethal dose ($LD_{50}$) in male rats was found to be about 10 mg./kg. for the L-form of PAM while it was 30 mg./kg. for the D-isomer (Boyland and

Seeking, personal communication). The difference made it possible to increase the dose of D-isomer in some experiments with the aim of producing the same number of cells with chromosome injuries, and the same degree of growth inhibition in the Walker carcinoma as was found after the administration of the L-isomer. The data obtained suggests that 3 mg./kg. of D is equivalent to 1 mg./kg. of L in producing the same number of cells with chromosome injuries in the Walker tumour 24 and 48 hours after intraperitoneal injection. In order, however, to suppress the growth of a 24 hour tumour graft, it was necessary to increase the dose of D isomer to 5 times that of L-form.

Thus the various biological tests, when they are considered together, strongly suggest that the L-form of PAM is more active as a growth-inhibitor of some particular transplantable tumours than either the D or DL forms. The question naturally arises as to what are the differences between the isomers which could account for the differences in biological activity. If for instance there were a difference in the rate of transportation, excretion, etc. between the L and D forms, they might be absorbed differentially by the tumour cells. In the present investigation some differences have been noted which would suggest that the physical properties of the isomers may play a role. It was mentioned previously that the difference in the number of injured cells was greater when the two isomers were administered in arachis oil suspensions and not in the sodium salt form (Table III). Similarly at high dose levels the difference between the L and D forms in producing cell injuries in the Landschütz ascites tumour, was greater than at low dose level (Table V and Fig. 18).

TABLE V.—*Percentage of Anaphases with Fragments in Landschütz Ascites Treated with the Isomers of PAM.*

(100 dividing cells were counted in each cell).

| Dose (mg./kg.). | Isomeric form. | Per cent of cells with fragments. 24 hr. | 48 hr. | 72 hr. | 96 hr. | 120 hr. | 144 hr. |
|---|---|---|---|---|---|---|---|
| 8 | L | 64 | 85 | 81 | 76 | — | — |
| | D | 55 | 71 | 76 | 78 | — | — |
| 4 | L | 45 | 69 | 72 | 76 | 71 | 60 |
| | D | 42 | 79 | 67 | 61 | 66 | 51 |
| | DL | 40 | 73 | — | — | 46 | 65 |
| 2 | L | 42 | — | — | — | — | — |
| 1 | L | 30 | 38 | 44 | 40 | 38 | 20 |
| | D | 31 | 40 | 42 | 37 | 35 | 18 |
| 0·4 | L | 9 | 19 | 4 | 9 | 6 | 6 |

These few facts seem to suggest that the mustard derivative of phenylalanine may act in cell metabolism as the L-isomer : i.e. the D form must be racemised before it can interact with cellular enzymes or intermediates. It is, however, possible that the D-form of PAM is active as such, but owing to its stereochemical configuration the incorporation of this isomer into cellular metabolites is slow. Both processes would introduce a time lag : some loss would occur if racemisation were involved since this, would result in the lower biological activity of the D isomer observed in our experiments. The differences seen in the time of onset of mitosis suppression in a 1 day old Walker tumour graft after the administration of L and D isomers suggests that this may be the case.

SUMMARY

1. A nitrogen mustard derivative of phenylalanine inhibits the growth of certain experimental tumours in rats and mice.

2. The growth inhibition is initiated by injuries to the chromosome mechanism, which leads to the death of tumour cells.

3. Cells in malignant effusions are more sensitive to the drug than cells in the subcutaneous growing solid tumour.

4. The L-isomer is more effective as an inhibitor of the growth of the Walker carcinoma than the D or DL forms.

5. In producing chromosome injuries in the Walker tumour cells the L-isomer is 3 times more active than the D form and it is 5 times more effective as a growth inhibitor of this tumour than the D-isomer.

6. The difference in activity between the L and D or DL forms is greater when the drug is administered in arachis oil suspension.

7. The drug increases the survival time of mice inoculated with the Landschütz ascites tumours. The L-isomer was the most effective, prolonging life to 36·5 days as compared with 15·5 days for control mice. The D and DL-forms were less effective in this respect than the L-form.

8. In both the Walker carcinoma and the Landschütz ascites, the differences shown by the three isomers on growth inhibition becomes measurable 7 and 12 days respectively after administration of the drug.

The authors are greatly indebted to Professor A. Haddow for his help and to Professor F. Bergel and Dr. J. A. Stock for their assistance throughout the investigation which was supported by grants to the Chester Beatty Research Institute (Institute of Cancer Research ; Royal Cancer Hospital) from the British Empire Cancer Campaign, the Jane Coffin Childs Memorial Fund for Medical Research, the Anna Fuller Fund, and the National Cancer Institute of the National Institutes of Health, U.S. Public Health Service.

### REFERENCES

BAYREUTHER, K.—(1952) *Z. Naturf.*, **7**, 554.

BERGEL, F. AND STOCK, J. A.—(1953) *Rep. Brit. Emp. Cancer Campgn*, **31**, 6. (1954) *J. chem. Soc.*, 2409.

EISEN, M. J.—(1940) *Amer. J. Cancer*, **39**, 36.

HADDOW, A.—(1953) *Rep. Brit. Emp. Cancer Campgn*, **31**, 6.

KLEIN, G.—(1951) 'The production of ascites tumors in mice and their use in studies on some biological and chemical characteristics of neoplastic cells'. Uppsala (Almgnist and Wiksells).

*Idem* AND RÉVÉSZ, L.—(1953) *J. nat. Cancer Inst.*, **14**, 229.

KOLLER, P. C.—(1955) *Ibid.*, **15**, 1237.

*Idem* AND CASARINI, A.—(1952) *Brit. J. Cancer*, **6**, 173.

LARIONOV, L. F., SHKODINSKAJA, E. N., TROOSHSIKINA, V. I., KHOKHLOVE, A. S., VASINA, O. S. AND NOVIKOVA, M. A.—(1955) *Lancet*, ii, 169.

LUCK, J. M.—(1956) *Science*, **123**, 984.

TJIO, J. H. AND LEVAN, A.—(1954) *Acta Univ. lund.*, N.F. Avd. 2. Bd. 50. Nr. 15, p. 1.